# A rare low-grade myofibroblastic sarcoma in lower jaw with the resemblance to benign lesions

**DOI:** 10.1186/s12903-022-02381-1

**Published:** 2022-09-05

**Authors:** Martina C. Schwerzmann, Matthias S. Dettmer, Daniel Baumhoer, Tateyuki Iizuka, Valerie G. A. Suter

**Affiliations:** 1 Department of Oral Surgery and Stomatology, School of Dental Medicine, University of Bern, Freiburgstrasse 7, 3010 Bern, Switzerland; 2 Clinic for Oral and Maxillofacial Surgery, University Hospital Zurich, Zurich, Switzerland; 3 Institute of Pathology, University Bern, Bern, Switzerland; 4 Institute of Pathology, Klinikum Stuttgart, Stuttgart, Germany; 5 Bone Tumour Reference Center at the Institute of Pathology, University and University Hospital Basel, Basel, Switzerland; 6 Department of Cranio-Maxillofacial Surgery, Bern University Hospital, Inselspital, Bern, Switzerland

**Keywords:** Diagnostic challenge, Low-grade-myofibroblastic sarcoma, Malignant spindle cell tumor, Myofibroma

## Abstract

**Background:**

Low-grade myofibroblastic sarcoma (LGMS) is a rare solid infiltrative soft tissue tumor with a predilection for the head and neck region.

**Case presentation:**

We report the diagnostic steps of a fast-growing lesion of the lower left jaw in a 45-year-old otherwise healthy woman. A first biopsy and subsequent histopathological examination showed potential differentials of a benign myofibroma, benign nodular fasciitis or an LGMS. This diagnostic overlap was a challenge for the decision of the further treatment approach. The treatment consisted of a segmental en bloc resection of the mandible including the second premolar, first and second molar. Histopathological examination of the resected tumor confirmed an LGMS.

**Conclusion:**

The histopathologic resemblance of LGMS to a range of benign and reactive tumors may lead to misdiagnosis and mistreatment. The rarity of LGMS explains the lack of established treatment protocols. This case shows the importance of adequate clinical decisions, expertise in the histopathology of rare tumors and interdisciplinary exchange to achieve state-of-the-art patient management.

## Background

Low-grade myofibroblastic sarcoma (LGMS) is a rare fibroblastic/myofibroblastic malignant tumor, distinctly classified by the World Health Organization (WHO) [1]. It is characterized by the increased proliferation of myofibroblasts. The etiology of LGMS is unknown. This solid infiltrative soft tissue tumor occurs in various sites of the body with a predilection for the head and neck region, particularly the tongue followed by the mandible, neck, larynx, palate, maxilla and lips [2]. Patients’ mean age is 40 years, but it has been reported in all age groups [3]. Due to scarcity of published cases and the limited size of case series in the literature, it remains unknown if there is a gender predilection [4]. LGMS is mostly described as a slow-growing, indolent mass and it is therefore frequently misdiagnosed initially as a benign lesion. Destructive patterns may become apparent with the radiologic examination [5]. The histopathologic diagnosis is challenging due to the rarity of LGMS and morphologic resemblance to closely related benign lesions. The histologic appearance can vary from fasciitis-, fibromatosis- to fibrosarcoma-like patterns. LGMS has a high recurrence potential while metastatic spread occurs rarely [2].

## Case presentation

**Fig. 1:**
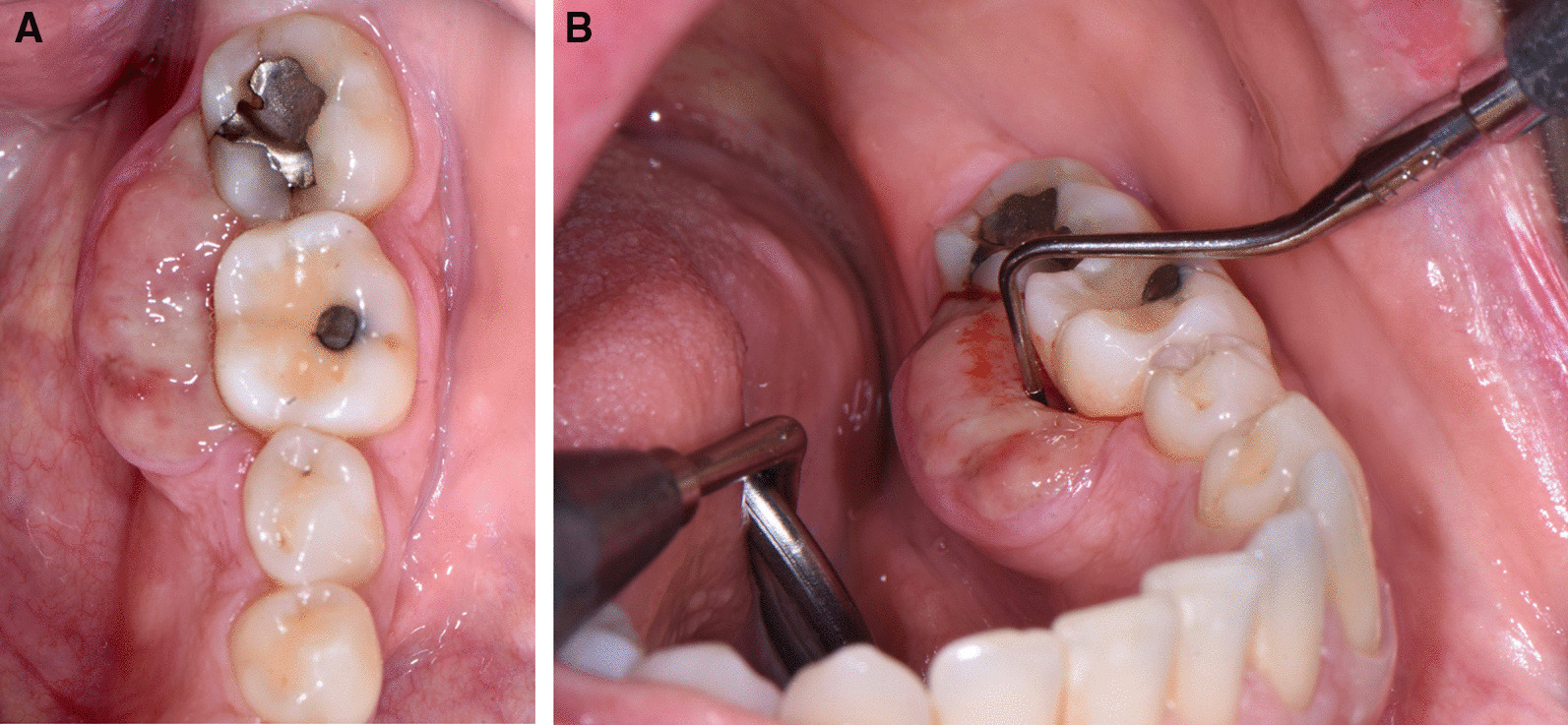
Intraoral photograph showing the tumor with the ulcerated surface in the left posterior mandible on the lingual side. **A**: occlusal view **B**: anterior and lingual view with a periodontal probe

**Fig. 2:**
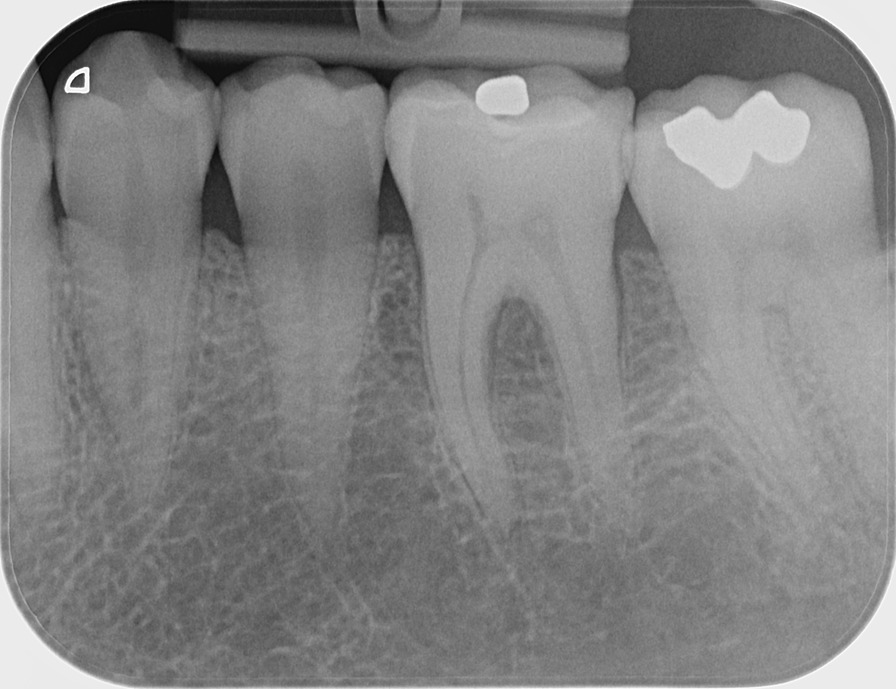
Apical radiograph with a dish-shaped radiolucency in left mandibular first molar region

**Fig. 3:**
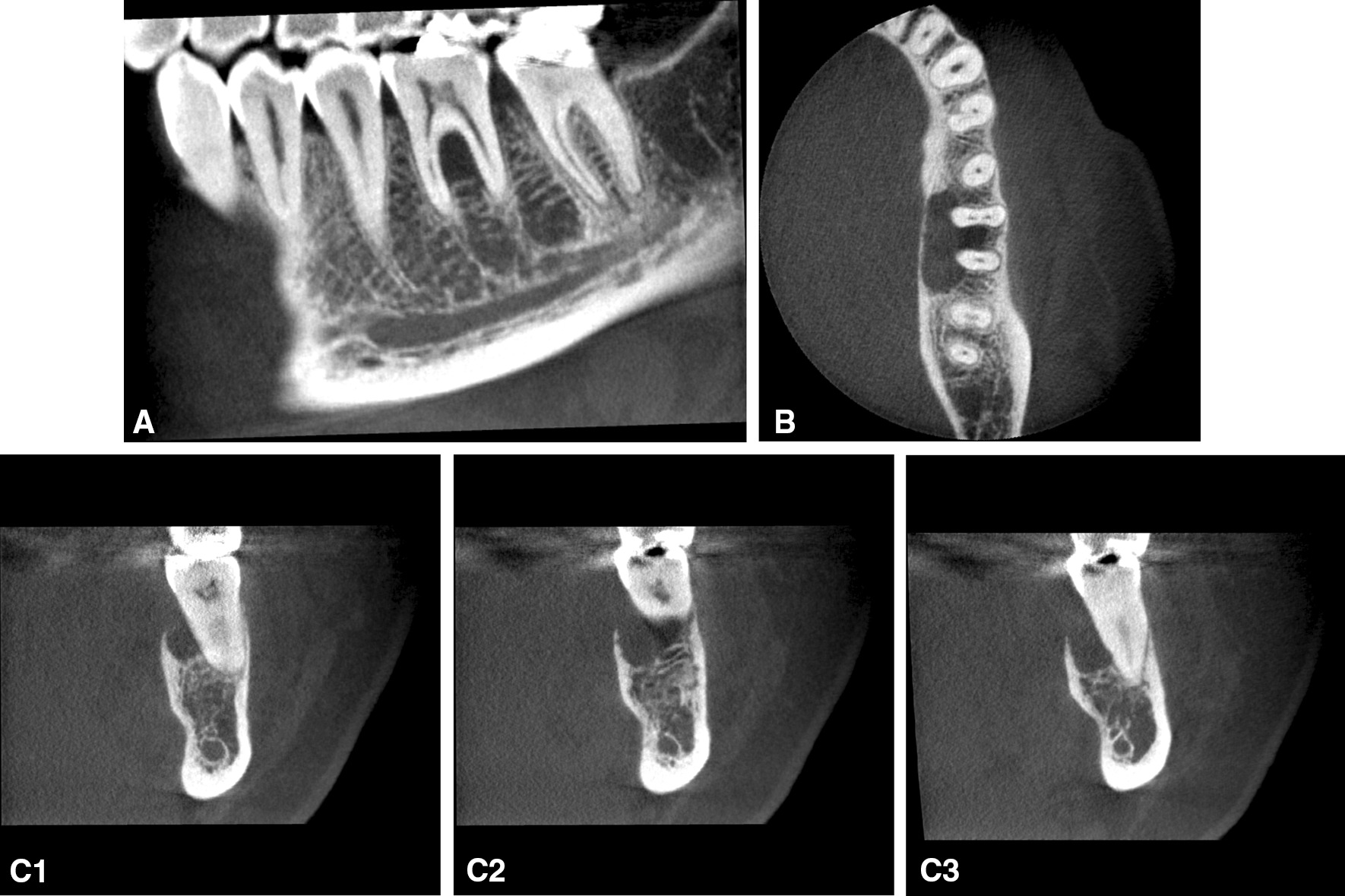
Cone beam computed tomography (Accuitomo 3D 170, Morita Corp, Kyoto, Japan) scan of the left mandible (full scan rotation 17.5 s, exposure setting 90 kV, 5.0 mA, field of view 6 × 4 cm). A radiolucency of the alveolar bone lingual and interradicular of the first left molar can be observed. The demarcation is partially unsharp and fenestrated to the crestal. (**A**: sagittal, **B**: axial, **C1-3**: coronal; **C1**: mesial root, **C2**: interradicular, **C3**: distal root)

A 45-year-old otherwise healthy female presented at the Department of Oral Surgery and Stomatology, University of Bern with a painful exophytic, lobulated tumor in the left posterior mandible on the lingual side. The patient had first noticed a lump two weeks ago and her dentist interpreted it as an epulis. Within 5 days, the tumor rapidly increased in size and was accompanied by pain. The patient experienced no fever, night sweats or weight loss. She denied any history of trauma. Her medical history was non-contributory and she had never smoked. The extraoral examination showed neither swelling nor facial asymmetry nor paresthesia. In the head and neck area, no clinical evidence of lymphadenopathy was observed. On clinical oral examination, the round-to-ovoid mass measured 12 × 20 mm in size and was firm to palpation, superficially ulcerated, and fixed to the underlying tissues (Fig. 1A). The first left mandibular molar showed an increased mobility, but not the premolars and the second molar. The examination with the periodontal probe showed normal pockets in the upper and lower jaw, except for the first lower molar (Fig. 1B). All teeth in the left posterior mandible were positive when testing with CO_2_ dry ice. The patient had a good oral hygiene. The periapical radiograph showed a bowl-shaped radiolucent area around the first molar (Fig. 2). Cone beam computed tomography (CBCT) demonstrated a radiolucent lesion on the lingual side of the first molar along and between the roots. The unilocular osteolytic area involved the marginal and lingual aspect of the bone and was fenestrated to the crestal side. The borders were ill-defined (Fig. 3). There were no signs of root resorption of the left first molar or adjacent teeth.

**Fig. 4:**
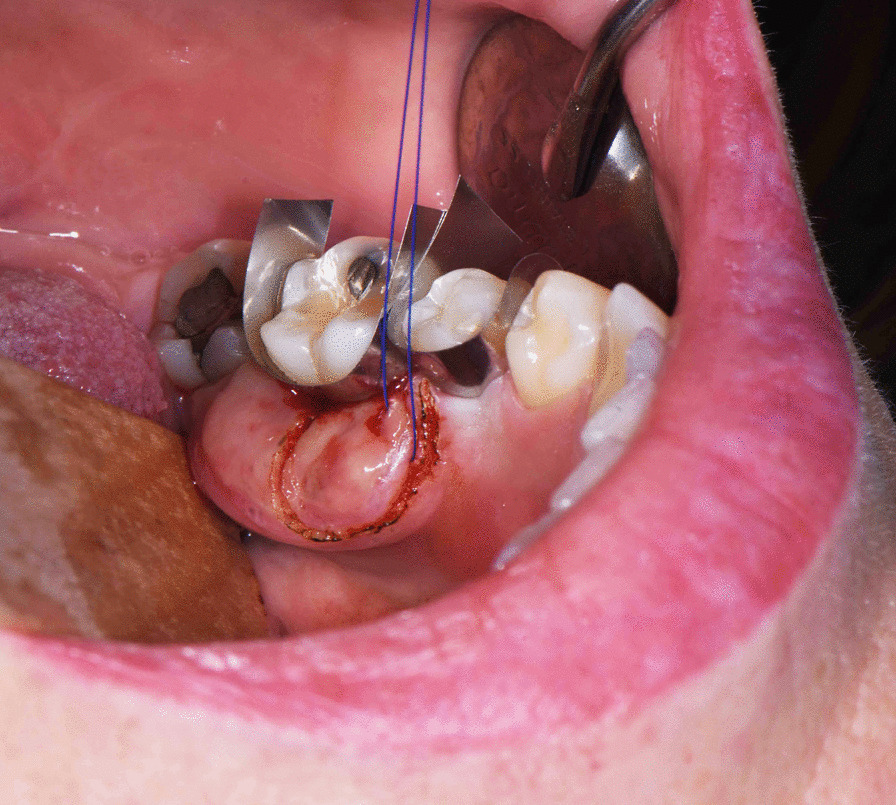
The representative incisional biopsy was performed with a CO_2_ laser (λ = 10.6 μm, Spectra DENTA Surgical Carbon Dioxide Laser, MAX Engineering Ltd., Gyeonggi-Do, Korea, char-free mode with frequency 140 Hz, pulse duration 400 µsec, pulse energy 33 mJ). The intraoral photograph is showing the demarcation of the incisional biopsy and the suture (Polyamid, 5–0) as orientation. The teeth were protected with metallic dies to avoid any iatrogenic damage with the laser beam

**Fig. 5:**
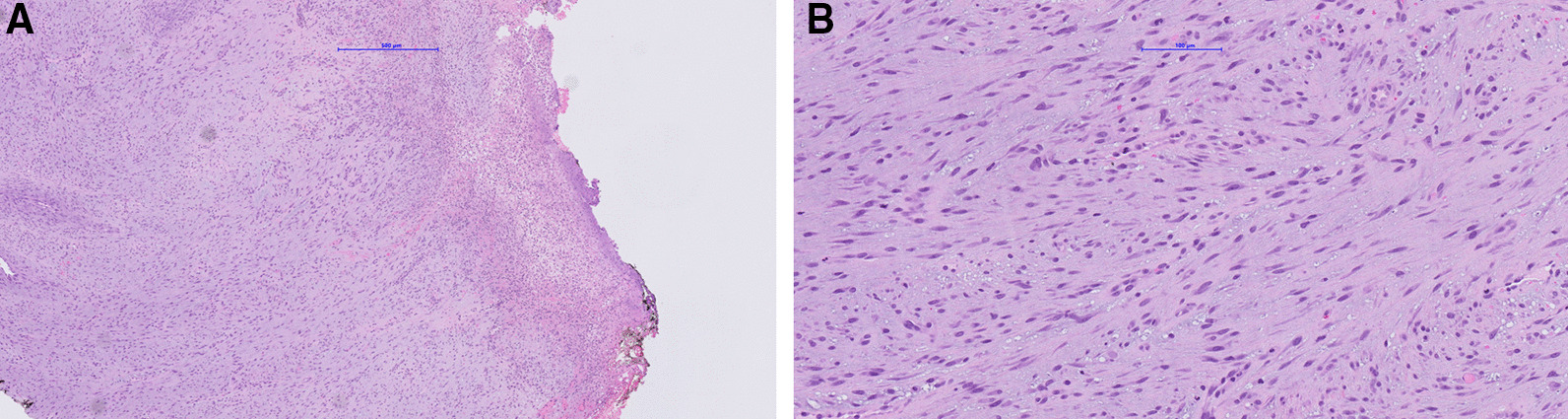
Photomicrograph (Nanozoomer S360, Hamamatsu) of the biopsy specimen with loosely arranged cells exhibiting a fasciculated and storiform growth pattern in a fibrous and myxoid background. In the lower half is squamous cell epithelium, whereas the majority of the specimen is ulcerated (A: hematoxylin and eosin stain, magnification 50x). High magnification shows cigar-like spindle cells with marginally hyperchromatic nuclei (B: magnification 200x)

**Fig. 6:**
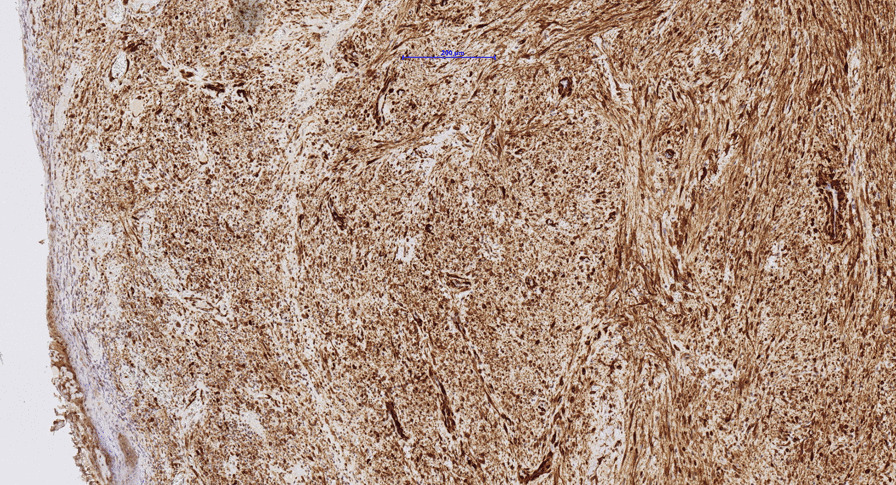
Photomicrograph (Nanozoomer S360, Hamamatsu) showing the positive immunohistochemistry for smooth muscle actin (SMA) of the same biopsy specimen as Figs. 4 & 5 (magnification 100x)

An incisional biopsy of the firm mass was performed with a carbon dioxide laser (Fig. 4). Histopathological examination showed a proliferation of haphazardly arranged spindle cells with high mitotic activity as well as a high degree of vascularization. A storiform and partially fasciculated architecture was found. The background was fibrous and showed partially myxoid aspects (Fig. 5A). Higher magnification revealed elongated spindle-shaped cells with longitudinal and oval-shaped, cigar shaped and slightly hyperchromic nuclei (Fig. 5B). The proliferation index Ki-67 was about 25–35%. Immunohistochemistry showed positivity for SMA, HHF35 and Calponin, but was negative for Caldesmon and Desmin (Fig. 6). As the diagnostic evaluation was challenging, the samples were sent to the DÖSAK (German-Swiss-Austrian Working Group of Maxillofacial Tumors) reference registry in Basel (Switzerland) for second opinion. The proposed differential diagnosis encompassed a benign lesion, an intermediate malignant transformation or a low-grade malignant process. The main differential diagnosis included myofibroma and LGMS, but the degree of atypia and the lack of a biphasic morphology favored LGMS.

**Fig. 7:**
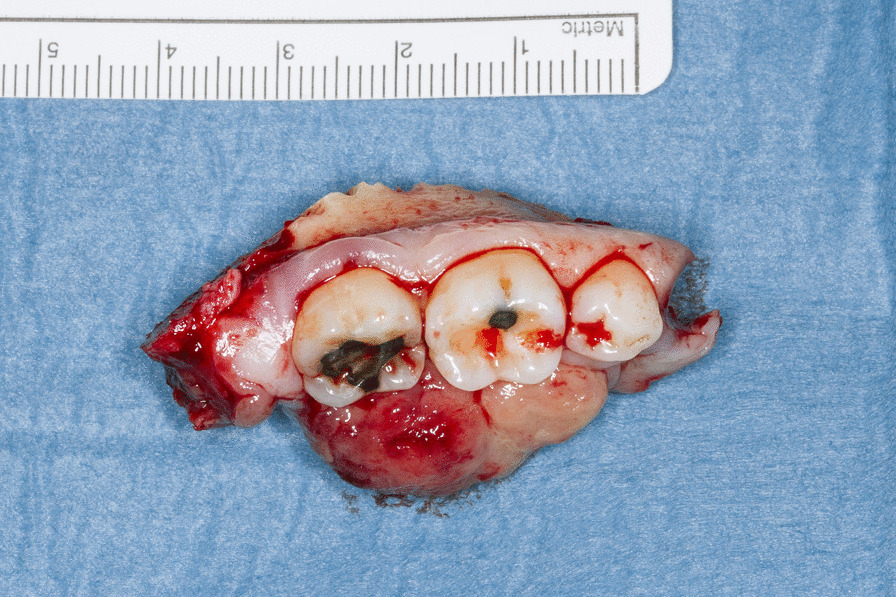
Fully resected tumor and surrounding bone and teeth after en bloc resection

**Fig. 8:**
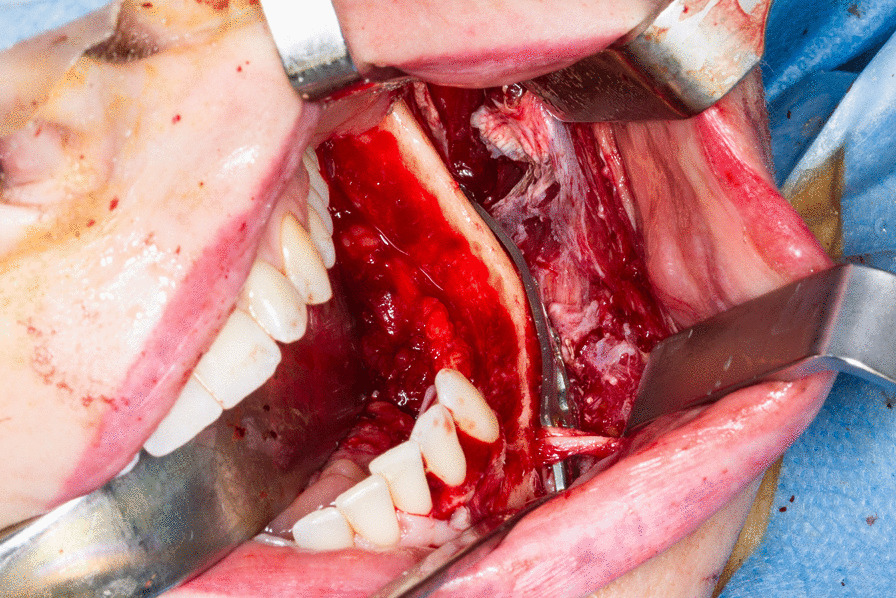
Left mandible after en bloc resection of the tumor and stabilized with a 3D-Grid plate for fracture prevention

**Fig. 9:**
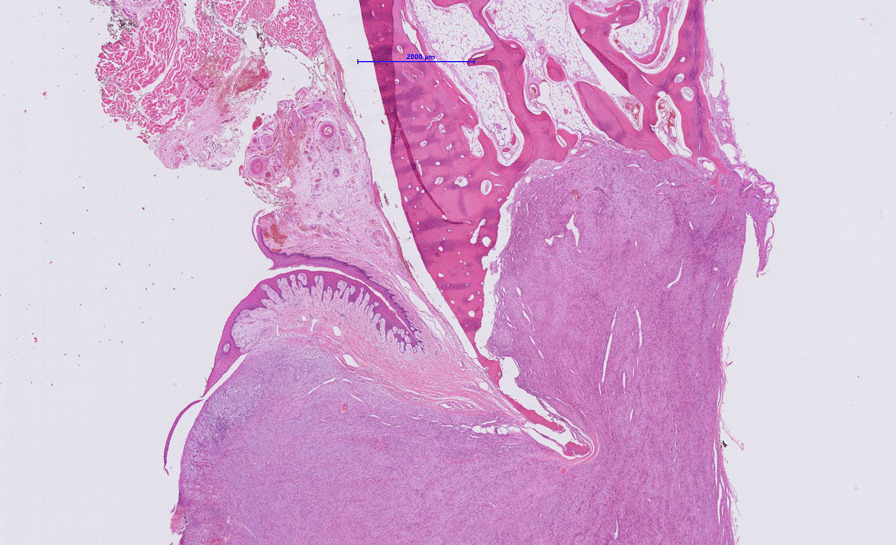
Broad infiltration of the mandible with bone destruction can be appreciated at low magnification in the photomicrograph (Nanozoomer S360, Hamamatsu), (hematoxylin and eosin stain, magnification 1.4x)

Consequently, the pathologists recommended tumor resection with subsequent histopathological evaluation. The planning of the next therapeutic steps took a possible malignant tumor into consideration. The treatment consisted of a segmental en bloc resection of the mandible including the second premolar, the first and second molar and was performed with a sufficiently large safety margin (Fig. 7). The horizontal border of the bone resection was set immediately above the mandibular canal. The inferior alveolar nerve was preserved. In connection with the tumor removal, the remaining mandibular bone was stabilized using a 3D-Grid Plate for fracture prevention (Fig. 8). Histopathological examination of the resected tumor showed an unencapsulated, well-circumscribed mass of numerous atypical spindle cells. The mitotic rate was 5/10 high power fields. Considering the superficial ulceration, the infiltration with destruction of the underlying compact bone as well as the morphology described, the diagnosis of LGMS was confirmed (Fig. 9). Resection margins were clear.

One year after tumor resection there were no signs of recurrence. To achieve vertical bone augmentation, transplants of autologous bone from the outer table were fixed with titanium screws to the left mandible, complemented with a mixture of autologous bone, blood, bone substitute particles and covered with a resorbable membrane.

## Discussion and conclusions

This case shows the challenging diagnostic and therapeutic decisions of a low-grade malignant spindle cell tumor with similarities to benign, reactive or other malignant tumors. After clinical and radiological examination, benign and malignant lesions were both possible. The differentials considered were a giant cell lesion of the jaw (GCLJ), a myofibroma, a nodular fasciitis, a squamous cell carcinoma, a lymphoma, or a metastasis. The presence of a rare tumor was also discussed. The quite well demarcated firm soft tissue mass had the appearance of a benign process, whereas its fast growth in conjunction with bony destruction and mobility of a single tooth were typical signs for malignancy. This clinical discrepancy of a possible benign or malignant lesion reinforced the importance of a biopsy. However, the histopathologic diagnosis was particularly difficult in the present case.

A common oral GCLJ is the peripheral giant cell granuloma (PGCG) or so-called giant cell epulis. Trigger factors such as dental trauma or local irritation such as chronic infections, dental plaque or food impaction as well as poorly adapted margins of restorations or orthodontic therapy have been discussed [3, 6]. Erosion of the adjacent alveolar bone has been described, but the presence of a crater-like osteolytic lesion in the radiograph was not typical for a PGCG. After histopathological examination of the first biopsy specimen a GCLJ was ruled out.

Another reactive process is nodular fasciitis, first described by Konwaler in 1955 and often found in the subcutaneous fascia of adults aged between 20 to 40 years [1, 7]. The head and neck region is affected in 20% of all cases, but lesions in the oral cavity are rare. [8]. The histopathology of a nodular fasciitis shows a variably cellular fibroblastic and myofibroblastic proliferation, arranged in short irregular fascicles, growing in a so-called cell culture pattern, sometimes with a high mitotic index. The presence of prominent vasculature and extravasated red blood cells can be helpful in differentiation from other myofibroblastic lesions. Clinically and histologically, nodular fasciitis can be misdiagnosed as a sarcoma. A rearrangement of the USP6 gene is commonly found and can be of great diagnostic help in difficult cases [1, 3]. The clinical context of the case presented here fitted well with a nodular fasciitis: a rapidly growing tumor-like mass, increasing pain and no evidence of systemic disease. The clinical appearance alone was, however, not reliable for diagnosis [9]. Nodular fasciitis is self-limited and often a spontaneous regression can be observed. Incisional biopsy with histopathological examination remains essential for the diagnosis and subsequent procedure.

The myofibroma is a rare benign soft-tissue neoplasm of the head and neck region and affects mostly the mandible, the tongue or the buccal mucosa. A genetic predisposition and factors such as trauma and injury have been discussed in the literature [10, 11]. The myofibroma is a fairly indolent slow-growing firm mass with lobulated surface, but cases with rapid and aggressive growth have also been described. It can present intraosseous as well-defined, unilocular radiolucency, mainly in the posterior mandible [12]. Not in accordance with the present case is that 75% of the patients are younger than 20 years of age [11]. Histopathologically, a myofibroma is an unencapsulated, well-demarcated biphasic lesion composed of myofibroblasts. Tumor cells are spindle shaped with scant cytoplasm and sometimes showing a zoning phenomenon [10].

The most frequent malignant tumor affecting the oral cavity is in 90% of cases a squamous cell carcinoma (SCC) [13, 14]. Predilections sites for SCC in the oral cavity are the tongue and floor of the mouth, but all sites can be involved, including the gingiva and alveolar ridge. The prevalence of SCC on the gingiva has been reported in 14% [15] in a multicenter study including populations from Canada, Korea, Iran, Taiwan and Thailand to 32% [14] in a population from Lagos in Nigeria. The clinical findings of the present case with rapid growth of a tumor mass, superficial ulceration, as well as the infiltration of the alveolar bone were suspicious for an SCC. Atypical, however, was the mesio-distal symmetrical shape of the tumor mass and the intact mucosa to the caudal. The absence of risk factors such as tobacco and alcohol use as well as the age of the patient were also considered as unusual.

Lymphoma are the third most common malignancies in the oral cavity and the diffuse large B cell lymphoma not otherwise specified (DLBCL NOS) is most often diagnosed. DLBCL NOS can arise in patients without risk factors and 40% are extranodal. A rapid growing soft tissue tumor typically located at the palate or the gingiva or alveolar ridge is a typical sign. Some cases involving the alveolar bone have been described in the literature [16, 17].

Another consideration was the presence of an intraoral metastasis. In early stages, mucosal metastases present as exophytic, most often on the gingiva. Jawbone metastases are more frequently found in the mandible than the maxilla and the most common site is the molar area. Overall, metastatic dissemination is rarely found in the oral cavity [18] and the presented patient had no cancer history.

The histopathologic diagnosis was essential, but also difficult in the present case. Potential differential diagnoses like a PCLJ, an SCC or a lymphoma were easily ruled out histopathologically, whereas myofibromas, nodular fasciitis and an LGMS show a significant morphologic overlap. After the first biopsy this overlap and potential differential diagnoses were a challenge for the subsequent treatment approach. In the case of a benign tumor or reactive lesion, a highly invasive therapy should be avoided to minimize the patient’s morbidity and subsequent impact on quality of life. On the other hand, overlooking and not rapidly and adequately treating a malignant tumor can be fatal. Furthermore, there is a lack of immunohistochemical and molecular markers that differentiate a myofibroma from an LGMS with certainty. Chromosomal imbalances have been described in myofibroblastic sarcoma and, in a small case series, these genetic aberrations were occurring more often compared to nodular fasciitis [19]. However, the methods of detection have their limitations and it is not an established method used to identify malignancy of a tumor. The histopathologic resemblance of LGMS to a myofibroma and fibromatosis is often likely a source of diagnostic confusion. Furthermore, the possibility of a lack of experience of the clinician and/or the pathologist due to the rarity of these pathologies further complicates the matter [20].

In the present case, the amount of cellular atypia together with the locally aggressive and symptomatic growth (bone erosion, surface ulceration), were very suspicious for an LGMS. For this reason, a more radical treatment approach with extraction of teeth and adequate bone resection was suggested to the patient and subsequently performed by the maxillofacial surgeon. The histopathological analysis of the resected specimen showed the invasive growth of the tumor into the bone and together with the high mitotic rate confirmed the diagnosis of a LGMS. The margins of resection were tumor free.

The complete clinical picture of LGMS remains unclear. Often LGMS is a slow-growing and indolent tumor [4, 21, 22]. This is in contrast to our case. It is assumed that LGMS occurs far more often than the scarcity of reported cases might portray. While most studies reported the head and neck region, including oral cavity, as predilection sites, the incidence of LGMS in other regions of the body could be higher than expected as shown by one of the latest studies [23].

Wide tumor excision including surrounding tissue is the suggested therapy. A recent case series showed the association between local recurrence and the tissue invasion of LGMS as well as the surgical method. Resection margins > 2 cm are suggested to minimize the risk of recurrence [24]. However, wider safety margins are often more problematic in the oral cavity than in other parts of the body, such as the extremities. A histopathological confirmation of tumor-free margins is requested and ideally already available during the surgery, but does not exclude recurrence [23, 25]. Some authors have recommended an adjuvant chemotherapy and radiotherapy. The latter is controversial as it is supposed to even induce poorer prognosis, with a reported recurrence rate of 18.8% following surgery only and 71.4% after surgery and radiotherapy [2]. Definitive treatment criteria, treatment protocols and requirements are still undetermined and require further research.

While the potential for recurrence of LGMS is high, the risk of metastasis is low [21, 22, 24]. In an earlier case series, only one patient with multiple distant soft-tissue, intraosseous and pulmonary metastases after a long time interval was described. Thus, it seems appropriate to classify these neoplasms as malignancy [22]. However, a more recent case series observed a higher rate of metastasis than earlier studies. It could also be assumed, that recurrent cases are more prone to present as intermediate and high-grade, but this has not been observed [25].

This case shows the diagnostic and therapeutic challenge of a rare low-grade myofibroblastic sarcoma and the team approach of clinicians and pathologists alike in order to achieve state-of-the-art patient management.

## Data Availability

Not applicable.

## Abbreviations

LGMS: Low-grad myofibroblastic sarcoma
WHO: World Health Organization
GCLJ: Giant cell lesion of the jaw
PGCG: Peripheral giant cell granuloma
SCC: Squamous cell carcinoma
CBCT: Cone beam computed tomography
DLBCL NOS: Diffuse large B cell lymphoma not otherwise specified
DÖSAK: German-Swiss-Austrian Working Group of Maxillofacial Tumors
